# First reported Canadian case of *Trichophyton mentagrophytes* genotype VII infection among men who have sex with men (MSM)

**DOI:** 10.14745/ccdr.v51i09a05

**Published:** 2025-10-09

**Authors:** Tatiana Lapa, Anna Banerji, Julianne Kus, Kendall Billick

**Affiliations:** 1 Division of Dermatology, Department of Medicine, University of Toronto, Toronto, ON; 2 Tropical Disease Unit, Division of Infectious Diseases, University Health Network, Toronto General Hospital, Toronto, ON; 3 Department of Laboratory Medicine and Pathobiology, University of Toronto, Toronto, ON; 4 Public Health Ontario, Toronto, ON

**Keywords:** *Trichophyton mentagrophytes* genotype VII, tinea genitalis, sexually transmitted infection

## Abstract

Over the past 20 years, *Trichophyton mentagrophytes* (*T. mentagrophytes*) infections affecting the genital and pubic regions, with suspected sexual transmission, have been increasingly reported in South Asia and Europe. The first case in the United States was reported in 2024. We describe the first confirmed case of *T. mentagrophytes* genotype VII infection causing Majocchi granuloma in a Canadian male who had recently travelled to Mexico, with suspected sexual transmission. Raising awareness among healthcare professionals is critical for early diagnosis and preventing long-term sequelae. Tinea corporis presenting with deep lesions in the pubogenital region and not responding to topical medications should prompt consideration of sexually transmitted fungal infection and extended testing including molecular identification by DNA sequencing of fungal cultures.

## Introduction

*Trichophyton mentagrophytes* is a zoophilic dermatophyte, a fungal organism that primarily infects animals but can occasionally infect humans, causing superficial fungal infections of the skin and its appendages. *Trichophyton mentagrophytes* genotype VII (TMVII) is a recently identified genotype, strongly associated with sexual transmission, particularly among men who have sex with men (MSM). Cases have been reported from Europe, South Asia, Australia, Africa and the United States (US) (1–14). Comparative analyses of cases from these regions (**Table 1**) suggest a predominance of pubogenital tinea presentations among MSM, often associated with international travel or sexual transmission. A related species called *T. indotineae* (formerly known as *T. mentagrophytes* type VIII) is known to be circulating in Canada (15,16), but this is the first report, of TMVII. Notably, fungal sexually transmitted infections (STIs), including TMVII, are not currently reportable to public health and are absent from the Public Health Agency of Canada’s *Sexually transmitted blood-borne infections: Guides for health professionals* (17). This gap highlights the need for awareness and further research into their prevalence, transmission dynamics, and public health impact.

**Table 1:** Global cases of dermatophytosis involving *Trichophyton mentagrophytes*, 2001–2024

Country/region	Year of reports	Population affected	Place of possible infection	Mode of transmission	Clinical features	Reference
Spain	2001	Female commercial sex worker	Spain	Sexual transmission	Tinea cruris	Otero *et al.*
Nigeria	2002	Female sex worker	Nigeria	Sexual transmission	Tinea genitalis	Bakare *et al.*
Germany	2001	Female	Germany	Contact with infected ferret	Tinea corporis, Tinea genitalis	Beckheinrich *et al.*
Seoul, South Korea	2005	Female	South Korea	Contact with infected dog	Majocchi granuloma	Chang *et al.*
Denmark	2009	Heterosexual couple	Spain	Sexual transmission	Tinea gladiatorum, Tinea genitalis	Molenberg *et al.*
Switzerland, Zurich	2014	Heterosexual females (n=2) and males (n=5)	South-East Asia	Sexual transmission	Tinea genitalis	Luchsinger *et al.*
Bulgaria	2015	Female	Bulgaria	Unknown	Tinea genitalis, Majocchi granuloma	Bakardzhiev *et al.*
Germany	2016	Females (n=19) and males (n=11)	Austria, Germany, prior travelling to South Asia and Thailand	Close contacts with infected animals, sexual transmission	Tinea genitalis	Ginter-Hanselmayer *et al.*
Germany	2017	Heterosexual male	Thailand	Sexual transmission	Tinea barbae profunda	Wendrock-Shiga *et al.*
Australia	2017	Male	South-East Asia (Thailand)	Sexual transmission	Tinea genitalis, Majocchi granuloma	Gallo *et al.*
Germany	2017	Female	Egypt	Unknown	Tinea genitalis	Nenoff *et al.*
France, Paris	2021–2022	Male heterosexual and MSM (n=12)	Germany, France, Slovenia, Spain, India	Sexual transmission	Tinea barbae, Tinea genitalis, Majocchi granuloma	Jabet *et al.*
United States, NY	2024	MSM (n=4)	United States	Sexual transmission	Tinea faciei, Tinea genitalis, Tinea glutealis	Zucker *et al.*
Germany	2001	Female	Germany	Contact with infected ferret	Tinea corporis, Tinea genitalis	Beckheinrich *et al.*
Seoul, South Korea	2005	Female	South Korea	Contact with infected dog	Majocchi granuloma	Chang *et al.*
Nigeria	2002	Female sex worker	Nigeria	Sexual transmission	Tinea genitalis	Bakare *et al.*
United States, NY	2024	MSM (n=4)	United States	Sexual transmission	Tinea faciei, Tinea genitalis, Tinea glutealis	Zucker *et al.*
United States	2024	MSM (n=1)	Europe (Greece, England) and United States	Sexual transmission	Tinea corporis, Tinea cruris, Tinea genitalis	Caplan *et al.*

Abbreviations: MSM, men who have sex with men; NY, New York

## Current situation

**Figure 1:**
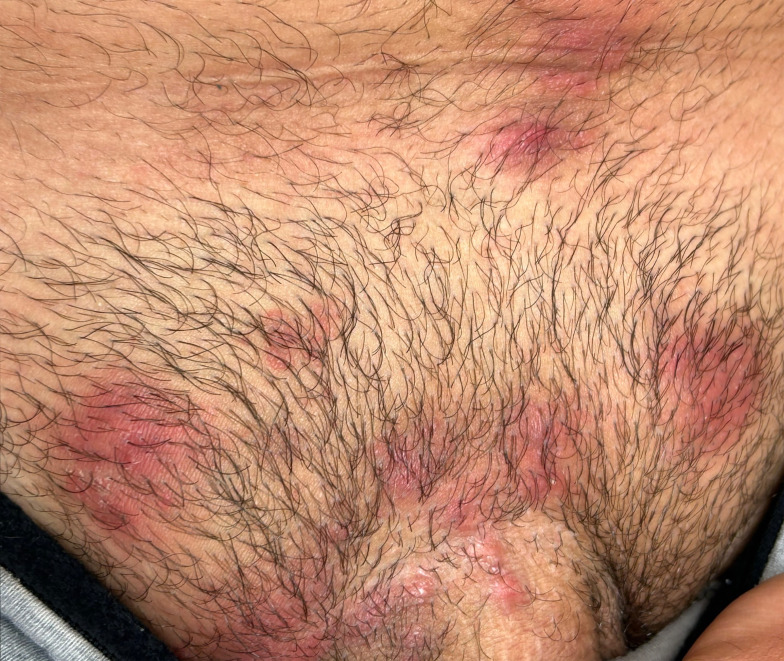
Multiple red papules, plaques, and subcutaneous nodules in the inguinopubic region of a male patient diagnosed with *Trichophyton mentagrophytes* genotype VII infection

A Canadian male in his 30s presented to the emergency room in May 2025 with a two-month history of a pruritic and progressive rash involving his arms and inguinal region (**Figure 1**). The rash began at the end of March, two weeks after returning from a two-week trip to an all-inclusive resort in Puerto Vallarta, Mexico. He was distressed because he had seen multiple doctors; his symptoms and rash persisted despite clotrimazole, betamethasone dipropionate as well as several topical and systemic antibacterials. Referrals to the departments of infectious diseases and dermatology were requested. Although he initially denied new sexual partners, he later reported sex with two other male partners while in Mexico. There was no significant environmental exposure or animal contact. The department of infectious diseases considered tinea, including tinea incognito due to prior topical steroids, secondary bacterial infection and psoriasis. Skin scraping revealed fungal elements but the culture was negative. The department of dermatology diagnosed Majocchi granuloma given numerous, coalescing, bright red, subcutaneous nodules and non-fluctuant papules in the inguinopubic region. Two biopsies were obtained: the one for Hematoxylin and Eosin stain (H&E) revealed a superficial and deep dermal lymphoeosinophilic infiltrate with negative Periodic Acid-Schiff (PAS). The second was sent for mycology. The fungal stain was negative but the culture grew *Trichophyton* species. This was later identified as TMVII through DNA sequence analysis of the internal transcribed spacer (ITS) region. Tests for immune compromise, including HIV infection, were negative.

The patient was treated with topical ciclopirox olamine and oral terbinafine until the lesions resolved; this required 10 weeks of therapy due to the involvement of hair follicles and deeper dermal layers, characteristic of Majocchi granuloma. The patient was advised not to shave the pubic region to prevent inoculating other parts of his body. In addition, he was advised to abstain from sexual relations until the lesions had fully resolved to avoid further transmission. Partner notification was suggested to raise awareness of potential exposure and to monitor for symptoms, such as pruritus or signs such as erythema or rash in the genital area. Our patient reported sex with two other partners with whom he maintained communication. Given that asymptomatic testing for fungal infections is not currently recommended and no chemoprophylaxis is available for sexually transmitted fungal infections, we advised that should these contacts develop symptoms or signs, they should abstain from further sexual relations and seek medical care.

## Conclusion

Dermatophytes are the primary cause of superficial fungal infections in humans and animals. Among these, *T. mentagrophytes* is a zoophilic species that primarily infects rodents, cattle and domesticated animals but can also infect humans, often through direct or indirect contact with an infected host (4,18). While the infection typically manifests as superficial tinea, it can present as a deep infection, such as Majocchi granuloma, particularly in immunocompromised individuals.

Tinea genitalis, or pubogenital tinea, is a rare form of dermatophytosis that directly involves the genitals and pubic region, in contrast to tinea cruris, which primarily affects the inguinal folds, upper inner thighs and buttocks. This condition is often seen in warm, humid climates and is most commonly caused by *Trichophyton* species, including *T. rubrum*, *T. interdigitale* and *T. mentagrophytes*. The infection is usually transmitted through autoinoculation, though sexual transmission has also been reported.

*Trichophyton mentagrophytes* genotype VII is a recently identified variant that is strongly associated with sexual transmission. Most reported cases involve MSM, with a few cases among heterosexual partners (4). Sexually transmitted TMVII has been increasingly reported in MSM communities, particularly in South Asia and Europe (2).

The first documented cases of tinea genitalis occurred in 2001, involving female sex workers in Spain (5). Since then, cases have been reported across Europe, Asia and, more recently, in the US. In 2023, the first case of TMVII was identified in a young male in the US with tinea genitalis and glutealis, suspected to be sexually transmitted (1).

Our patient, who is the first documented case in Canada, presented with a similar infection but involved deeper hair follicles and dermis, which is called Majocchi granuloma. Although the patient is immunocompetent, microtrauma may have predisposed him to the infection. Both *T. mentagrophytes* and *T. interdigitale* have been increasingly reported, yet the accurate identification of *Trichophyton* to the species level can be challenging especially due to evolving taxonomic assignments based on new understanding of genomic relationships (18). The *T. mentagrophytes* complex is now differentiated into *T. mentagrophytes*, which is zoophilic and associated with more inflammatory dermatophytosis in humans, and *T. interdigitale*, which is anthropophilic and primarily causes non-inflammatory tinea unguium and tinea pedis. While there are no commercial PCR assays that can distinguish between *T. mentagrophytes* and *T. interdigitale*, molecular markers, specifically sequencing the ITS region of fungal DNA, are used for accurate strain identification (18,19).

According to the nomenclature proposed by Nenoff *et al.* (18), the ITS phylogenetic tree includes *T. mentagrophytes* and *T. interdigitale* genotypes III (strains from animal hosts), III* (strains from soil), IV, V, VII, VIII and IX. *Trichophyton mentagrophytes* genotype VIII has been reclassified as a new species, *T. indotineae*, which is an emerging pathogen. Molecular analysis reveals that while *T. mentagrophytes* and *T. interdigitale* are difficult to distinguish from each other, they are clearly different from *T. indotineae*, which is known for human-to-human transmission, severe infections and a propensity for antifungal resistance to both terbinafine and fluconazole (15,20).

Accurate identification of *Trichophyton* species is critical, especially given the emergence of TMVII and *T. indotineae*. Traditional methods such as fungal scraping, culture and phenotypic identification may not be sufficient to distinguish between all *Trichophyton* species. Molecular techniques, particularly sequencing of the fungal ITS region, are currently essential for accurate identification and may be warranted in some cases. It is important to note that DNA sequencing of dermatophytes is not routinely performed and may need to be specifically requested.

Reported cases highlight the need to consider fungal STIs in patients with atypical presentations, especially in the genital area. This case emphasizes the potential for global spread and the importance of considering travel history in patients with similar symptoms. It contributes to the growing evidence linking TMVII with STIs. The global spread of this genotype underscores the need for clinicians to be vigilant in identifying and managing such cases, particularly in patients with relevant travel histories and sexual activity within at-risk communities.

This is the first reported case of sexually transmitted TMVII infection in Canada. The case highlights the need for heightened awareness among healthcare providers regarding the potential for sexually transmitted fungal infections, especially in patients with atypical tinea presentations involving the pubogenital region. Accurate diagnosis through molecular identification is essential for effective management. This case also underscores the importance of considering longer treatment durations for deep-seated infections, such as Majocchi granuloma, which require systemic antifungal therapy. Partner notification remains a critical component of care, raising awareness of potential exposure, encouraging medical evaluation if symptoms and signs develop, and especially abstinence until diagnosis and definitive therapy to prevent further spread. Sexually transmitted fungal skin infections are neither reportable to public health, nor covered in the Public Health Agency of Canada’s *Sexually transmitted and blood-borne infections: Guides for health professionals* (17). This underscores the importance of enhanced surveillance and public health initiatives in raising awareness and educating clinicians about these rare but impactful conditions. Finally, collaboration between clinicians, laboratories, and public health authorities are vital to improve detection, management, and prevention of such infections.

## References

[1] Caplan AS, Sikora M, Strome A, Akoh CC, Otto C, Chaturvedi S, Zampella JG. Potential sexual transmission of tinea pubogenitalis from Trichophyton mentagrophytes genotype VII. JAMA Dermatol 2024;160(7):783–5. 10.1001/jamadermatol.2024.1430 38837127

[2] Jabet A, Dellière S, Seang S, Chermak A, Schneider L, Chiarabini T, Teboul A, Hickman G, Bozonnat A, Brin C, Favier M, Tamzali Y, Chasset F, Barete S, Hamane S, Benderdouche M, Moreno-Sabater A, Dannaoui E, Hennequin C, Fekkar A, Piarroux R, Normand AC, Monsel G. Sexually transmitted Trichophyton mentagrophytes genotype VII infection among men who have sex with men. Emerg Infect Dis 2023;29(7):1411–4. 10.3201/eid2907.230025 37347803 PMC10310379

[3] Zucker J, Caplan AS, Gunaratne SH, Gallitano SM, Zampella JG, Otto C, Sally R, Chaturvedi S, O’Brien B, Todd GC, Anand P, Quilter LA, Smith DJ, Chiller T, Lockhart SR, Lyman M, Pathela P, Gold JA. Notes from the field: trichophyton mentagrophytes genotype VII — New York City, April–July 2024. MMWR Morb Mortal Wkly Rep 2024;73(43):985–8. 10.15585/mmwr.mm7343a5 39480750 PMC11527365

[4] Mølenberg D, Deleuran M, Sommerlund M. Connubial tinea gladiatorum due to Trichophyton mentagrophytes. Mycoses 2010;53(6):533–4. 10.1111/j.1439-0507.2009.01734.x 19682313

[5] Otero L, Palacio V, Vázquez F. Tinea cruris in female prostitutes. Mycopathologia 2002;153(1):29–31. 10.1023/A:1015257320824 11913763

[6] Wendrock-Shiga G, Mechtel D, Uhrlaß S, Koch D, Krüger C, Nenoff P. [Tinea barbae profunda due to Trichophyton mentagrophytes after journey to Thailand : case report and review]. Hautarzt 2017;68(8):639–48. 10.1007/s00105-017-4008-2 28616693

[7] Luchsinger I, Bosshard PP, Kasper RS, Reinhardt D, Lautenschlager S. Tinea genitalis: a new entity of sexually transmitted infection? Case series and review of the literature. Sex Transm Infect 2015;91(7):493–6. 10.1136/sextrans-2015-052036 26071391 PMC4680168

[8] Chang SE, Lee DK, Choi JH, Moon KC, Koh JK. Majocchi’s granuloma of the vulva caused by Trichophyton mentagrophytes. Mycoses 2005;48(6):382–4. 10.1111/j.1439-0507.2005.01147.x 16262873

[9] Barile F, Filotico R, Cassano N, Vena GA. Pubic and vulvar inflammatory tinea due to Trichophyton mentagrophytes. Int J Dermatol 2006;45(11):1375–7. 10.1111/j.1365-4632.2006.02793.x 17076734

[10] Ginter-Hanselmayer G, Nenoff P, Kurrat W, Propst E, Durrant-Finn U, Uhrlaß S, Weger W. [Tinea in the genital area : A diagnostic and therapeutic challenge]. Hautarzt 2016;67(9):689–99. 10.1007/s00105-016-3848-5 27488308

[11] Bakardzhiev I, Chokoeva A, Tchernev G, Wollina U, Lotti T. Tinea profunda of the genital area. Successful treatment of a rare skin disease. Dermatol Ther 2016;29(3):181–3. 10.1111/dth.12311 26555874

[12] Gallo JG, Woods M, Graham RM, Jennison AV. A severe transmissible Majocchi’s granuloma in an immunocompetent returned traveler. Med Mycol Case Rep 2017;18:5–7. 10.1016/j.mmcr.2017.07.003 28725545 PMC5502794

[13] Nenoff P, Schubert K, Jarsumbeck R, Uhrlaß S, Krüger C. Tinea genitalis profunda due to Trichophyton mentagrophytes after a journey to Egypt. Aktuelle Derm 2017;43(04):146–53. 10.1055/s-0043-106149

[14] Bakare RA, Oni AA, Umar US, Adewole IF, Shokunbi WA, Fayemiwo SA, Fasina NA. Pattern of sexually transmitted diseases among commercial sex workers (CSWs) in Ibadan, Nigeria. Afr J Med Med Sci 2002;31(3):243–7. 12751565

[15] McTaggart LR, Cronin K, Ruscica S, Patel SN, Kus JV. Emergence of terbinafine-resistant *Trichophyton indotineae* in Ontario, Canada, 2014-2023. J Clin Microbiol 2025;63(1):e0153524. 10.1128/jcm.01535-24 39584838 PMC11784349

[16] Avery EG, Ricciuto DR, Kus JV. Refractory tinea corporis or cruris caused by *Trichophyton indotineae*. CMAJ 2024;196(27):E940. 10.1503/cmaj.240408 39134313 PMC11318982

[17] Public Health Agency of Canada. Sexually transmitted and blood-borne infections: Guides for health professionals. Ottawa, ON: PHAC; 2025. [Accessed 2025 Jan 26]. https://www.canada.ca/en/public-health/services/infectious-diseases/sexual-health-sexually-transmitted-infections/canadian-guidelines.html

[18] Nenoff P, Verma SB, Vasani R, Burmester A, Hipler UC, Wittig F, Krüger C, Nenoff K, Wiegand C, Saraswat A, Madhu R, Panda S, Das A, Kura M, Jain A, Koch D, Gräser Y, Uhrlaß S. The current Indian epidemic of superficial dermatophytosis due to Trichophyton mentagrophytes-A molecular study. Mycoses 2019;62(4):336–56. 10.1111/myc.12878 30561859

[19] Tang C, Kong X, Ahmed SA, Thakur R, Chowdhary A, Nenoff P, Uhrlass S, Verma SB, Meis JF, Kandemir H, Kang Y, de Hoog GS. Taxonomy of the Trichophyton mentagrophytes/T. interdigitale species complex harboring the highly virulent, multiresistant genotype T. indotineae. Mycopathologia 2021;186(3):315–26. 10.1007/s11046-021-00544-2 33847867 PMC8249266

[20] Sonego B, Corio A, Mazzoletti V, Zerbato V, Benini A, di Meo N, Zalaudek I, Stinco G, Errichetti E, Zelin E. Trichophyton indotineae, an emerging drug-resistant dermatophyte: A review of the treatment options. J Clin Med 2024;13(12):3558. 10.3390/jcm13123558 38930086 PMC11204959

